# Analysis of Neural-BOLD Coupling Through Four Models of the Neural Metabolic Demand

**DOI:** 10.3389/fnins.2015.00419

**Published:** 2015-12-15

**Authors:** Christopher W. Tyler, Lora T. Likova, Spero C. Nicholas

**Affiliations:** Smith-Kettlewell InstituteSan Francisco, CA, USA

**Keywords:** fMRI, metabolic coupling, neural signal estimation, human brain, multimodal imaging, BOLD, local field potentials

## Abstract

The coupling of the neuronal energetics to the blood-oxygen-level-dependent (BOLD) response is still incompletely understood. To address this issue, we compared the fits of four plausible models of neurometabolic coupling dynamics to available data for simultaneous recordings of the local field potential and the local BOLD response recorded from monkey primary visual cortex over a wide range of stimulus durations. The four models of the metabolic demand driving the BOLD response were: direct coupling with the overall LFP; rectified coupling to the LFP; coupling with a slow adaptive component of the implied neural population response; and coupling with the non-adaptive intracellular input signal defined by the stimulus time course. Taking all stimulus durations into account, the results imply that the BOLD response is most closely coupled with metabolic demand derived from the intracellular input waveform, without significant influence from the adaptive transients and nonlinearities exhibited by the LFP waveform.

## Introduction

The goal of functional Magnetic Resonance Imaging (fMRI) is to estimate properties of the neural signals in the brain during the spectrum of activities controlled by the nervous system. However, the recorded fMRI signal is a response to the metabolic demands of supporting the nearby neural activity (Thompson et al., 2003, 2004). It is therefore important to understand as much as possible about the pathway coupling the recorded fMRI response to the dynamics of the neural activity giving rise to it. The theoretical development of the neural/BOLD coupling logic is based on that of Tyler and Likova (2011) although the present application to monkey joint fMRI/local-field-potential data is entirely novel.

### Neural/astrocyte coupling

**Figure 1:**
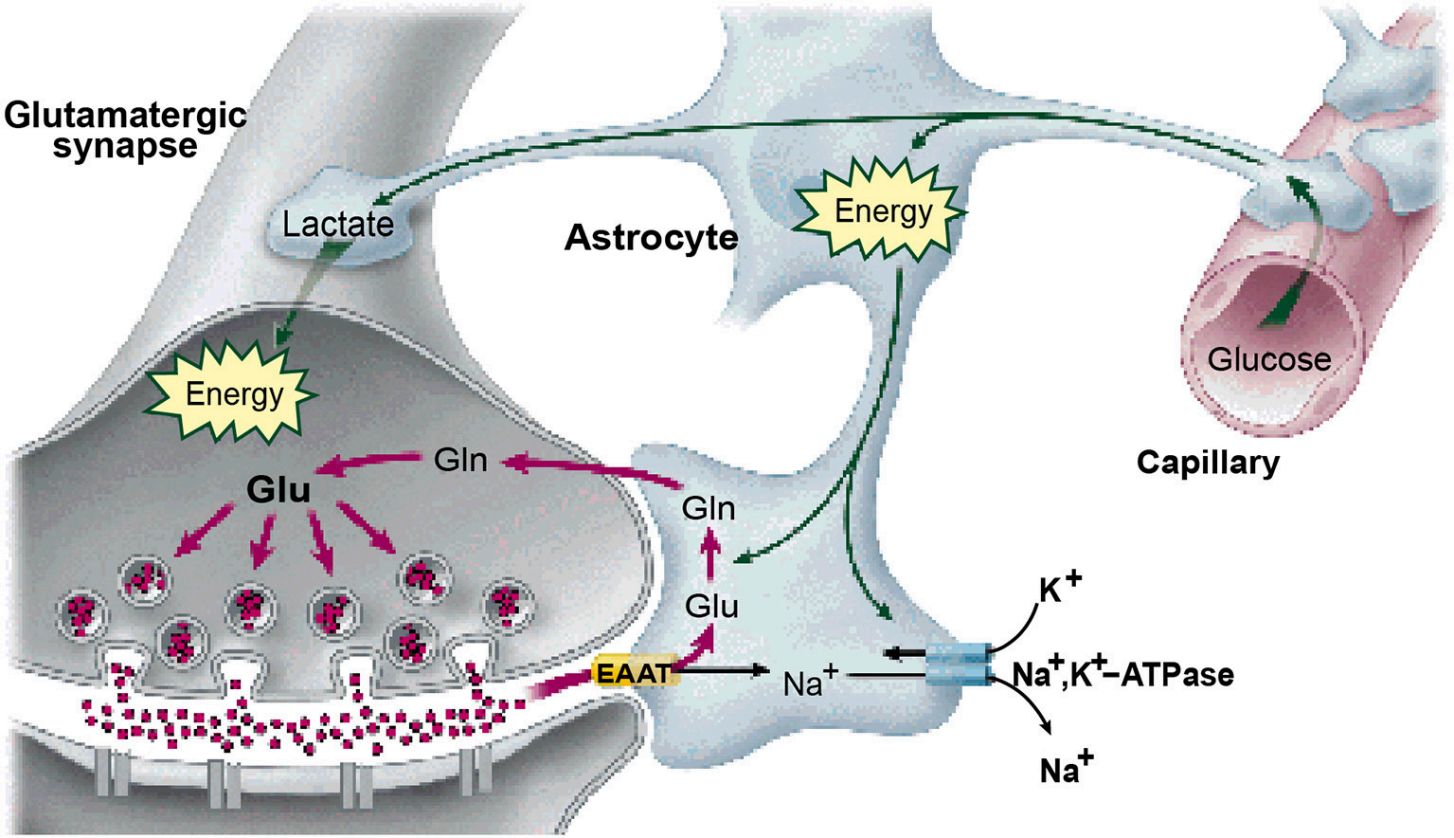
**The astrocytes as the substrate for the neurovascular coupling of the neural metabolism**. (From Magistretti, 2006, with permission).

It is widely accepted that the origin of the metabolic demand driving the blood-oxygen-level-dependent (BOLD) signal recorded in fMRI is the energetic load deriving from transmitter release at the synaptic inputs to each neuron (Logothetis, 2002, 2003; Logothetis and Wandell, 2004; Shmuel et al., 2006; Carmignoto and Gómez-Gonzalo, 2010). The transmitter release is tightly coupled to the activation of the post-synaptic receptors on the recipient cell membrane and consequently to the energetic demands of the synaptic activation of the transmitter molecules for future release, the majority of synapses being glutamatergic (Magistretti, 2006). The synaptic origin of the energetic demand driving the BOLD signal is thus coupled to the net transmitter signal impinging on the cells, and hence to the intracellular potential in these cells. The majority of these energetic demands are met by either by glycolysis of glutamate to glutamine in the neighboring astrocytes (Shank and Aprison, 1979; Wang and Floor, 1994; Bélanger et al., 2011; see Figure 1), or by oxidative phosphorylation from the neuronal mitochondria (Attwell and Laughlin, 2001; Hall et al., 2012; Pellerin and Magistretti, 2012).

It should be mentioned, however, that the existence of a direct interneuron pathway for vasodilation and vasoconstriction has also been proposed (Dirnagl et al., 1993; Ma et al., 1996), although the proportion of the effects specific to this direct pathway remain a matter of debate (Lindauer et al., 1996; Attwell et al., 2010). Indeed, we are unaware of any studies on this issue that provide evidence of interneuron control of vascular diameter having the fast (~5 s) time constant sufficient to account for the BOLD response dynamics in the human brain *in vivo*.

### Source of BOLD waveform variability

It is well known that there are substantial variations in the BOLD waveform in different cortical regions recorded during the same task (Handwerker et al., 2004, 2012; Fox et al., 2005), which have often been interpreted as due to variations in the local hemodynamics among cortical regions. Two points should be made in this regard. One is that differences in hemodynamics are largely attributable to differences in density of the arterial supply and draining veins overlying the cortical parenchyma (Handwerker et al., 2012), which indeed are expected to have different dynamics from the local capillaries within the cortex. However, this is an issue that can be addressed by accurate segmentation and the appropriate choice of voxel sizes to exclude extra-parenchymal signals and restrict the recorded BOLD responses to cortical space. To our knowledge, none of the papers evaluating the regional variations in BOLD waveform have implemented this strategy.

The other important point is that none of the studies of regional variations in BOLD waveform have assessed the role of neural variations in temporal waveform in this phenomenon. Neural waveform variations among neurons of different types and even the same types in different cortical regions are well-established (e.g., Hegdé and Van Essen, 2004, 2006). Such variations in the source signal can readily give rise to variations in the consequent BOLD waveforms, even on a different (longer) timescale (see Tyler et al., 2008; Tyler and Likova, 2011, 2014). Given this neurophysiological evidence, it is arbitrary and prejudicial to attribute all BOLD waveform variations purely to hemodynamics. There must be a neural component to this variation that needs to be acknowledged in all analyses of BOLD variations across regions.

Indeed, the logic of the known neural variations in neural signals poses the question whether *any* of the regional BOLD variation can be securely attributed to hemodynamic causes. All studies of regional BOLD variation to date have employed paradigms in which the BOLD responses are mediated by neural signals, whether in response to external stimulation or intrinsic neural interactions. As such, the BOLD responses were subject to the known functional variation of neural activity across regions of cortical specialization, and hence of potential temporal variation. Only if the neural signals were determined to be equal by direct measurement, or the BOLD signals across cortical regions were generated by a post-neural input, such as nitric oxide infusion in the region of the blood vessels, could the variation be convincing attributed to hemodynamic factors. However, in order to follow the first course, it is necessary to determine the aspect of the neural signals that is responsible for generating BOLD response dynamics, which is the topic of the present paper based on a novel analysis of simultaneously recorded local field potential (LFP) and BOLD signals from monkey cortex.

### Nonlinearity of the BOLD time course

**Figure 2:**
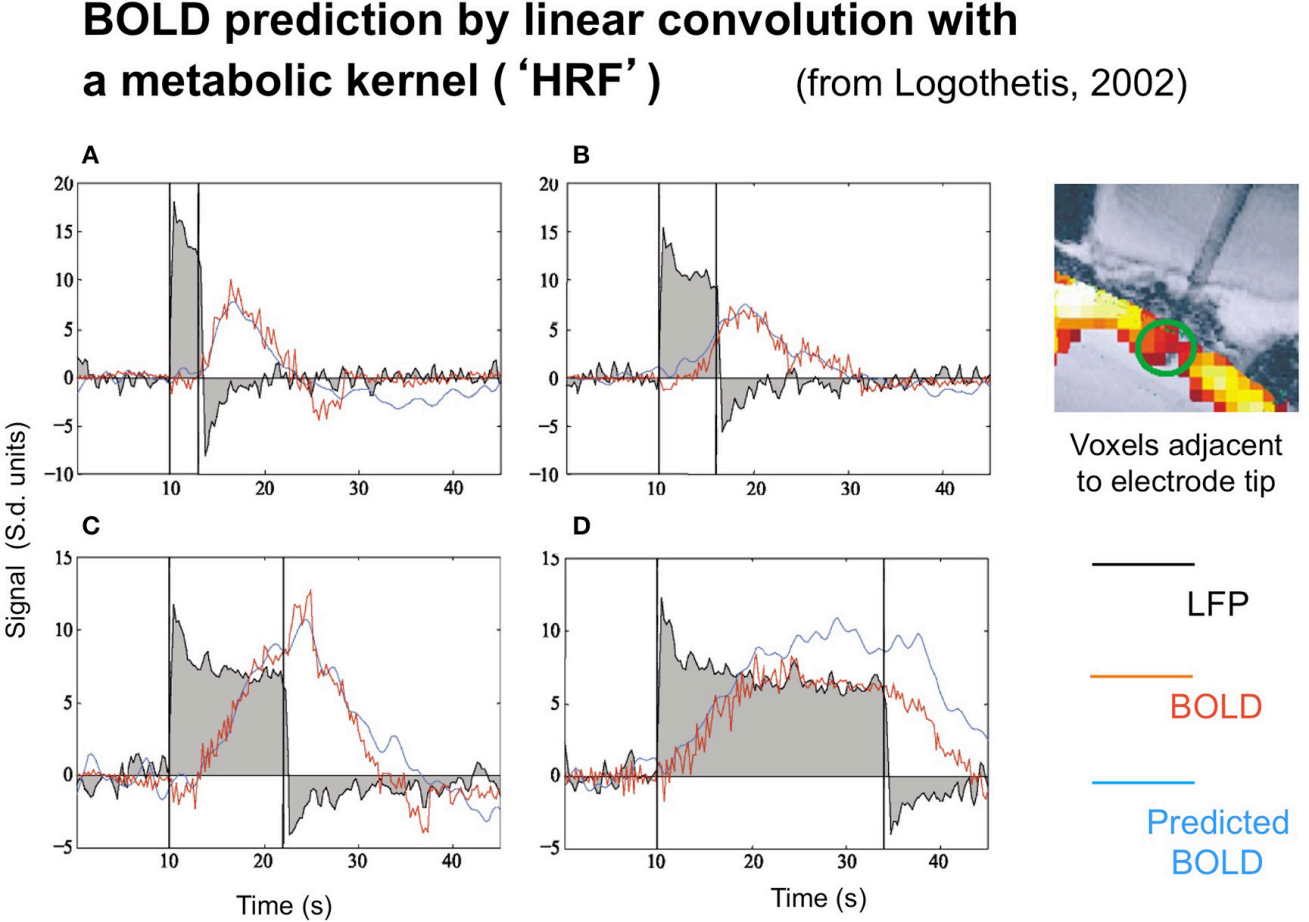
**Time course of the local field potentials (black trace), BOLD (red blue trace), and predicted BOLD (red trace) to a continuous dynamic stimuli (black rectangle) of 3, 6, 12, and 24 s duration (A–D, respectively from Logothetis, 2003, with permission)**. The prediction was generate by linear convolution of the recorded LFP signal with a hemodynamic response function (see Logothetis, 2002, for details).

The neural and BOLD response time courses were measured simultaneously to rotating checkerboards stimuli in a study by Logothetis (2003) in behaving monkeys. The neural time course was recorded in terms of the LFP, with the BOLD signal being recorded from 16 adjacent voxels (since the presence of the electrode prevented recording from the actual voxel containing it). Representative results are shown in Figure 2.

Two points are noteworthy. One is that the LFP timecourse (black curves) does not exactly match the stimulus timecourse (black box function) despite the author's efforts to do so by providing a continuously moving, high contrast target. The timecourse has the initial transient ubiquitously seen in single-unit recordings, followed by a sustained plateau that shows a gradual adaptation effect. The off-response shows a similar (inverted) transient, but only minimum evidence of the plateau. As a result, the overall LFP response is nonlinearly related to the stimulus in a manner that can be captured by a parallel-channel model of the sum of several component neural responses, but not by a serial model of convolution with any single form of temporal impulse response.

Logothetis' concern was not, however, with the linearity or otherwise of the LFP, but with its relation to the BOLD response. The BOLD time course was predicted on the basis of convolution of the recorded LFP waveforms with an estimated impulse response function. The function that provided a good fit for short duration stimuli, however, showed significant deviations from the measure data at long durations (Figure 2), predicting a substantially stronger BOLD response than was actually recorded at the longest duration, in particular.

This result implies that the neurometabolic coupling is not well-described by a linear convolution process, but has further *nonlinearities* built into it that need to be taken into account in an attempt to infer the neural signal on the basis of local BOLD response recordings.

The LFP recordings in Figure 2 make it apparent that the LFP waveform has a complex time course that can be approximated by two exponentials with time constants of about 1 s and >30 s, respectively. Relative to the usual time courses of neural transients, of about 50 ms these are remarkably prolonged neural processes on the time scale of the recorded BOLD signal from the same general region of cortex (blue trace).

The importance of this *adaptation effect* is emphasized by the fact that the recorded LFP signal does not fully match the predicted BOLD activation (red curve), and therefore a more comprehensive model is required, going beyond the standard General Linear Model (GLM) of convolution of a metabolic kernel with the stimulus time course. We note that a corresponding adaptation effect in the neural response to flickering stimulation was inferred by Pfeuffer et al. (2003) from the pattern of variations in BOLD response amplitude as a function of stimulus duration.

## Theoretical analysis

### Analysis of neural/BOLD coupling nonlinearities

The widespread utilization of the general linear model in fMRI analyses may be taken to imply that it is an adequate approximation to the BOLD signal behavior under typical recording conditions, but a detailed reveals some limitations of this model. As a starting point of the analysis, we have developed a specific model structure of the processes leading to the BOLD paramagnetic signal of fMRI recordings (Tyler and Likova, 2011). This model goes beyond the linear convolution analyses of Friston (1997) and Friston et al. (1998, 2000) in incorporating multiple forms of neural signal within each voxel and recognizing an explicit glial aspect to the metabolic coupling pathway.

**Figure 3:**
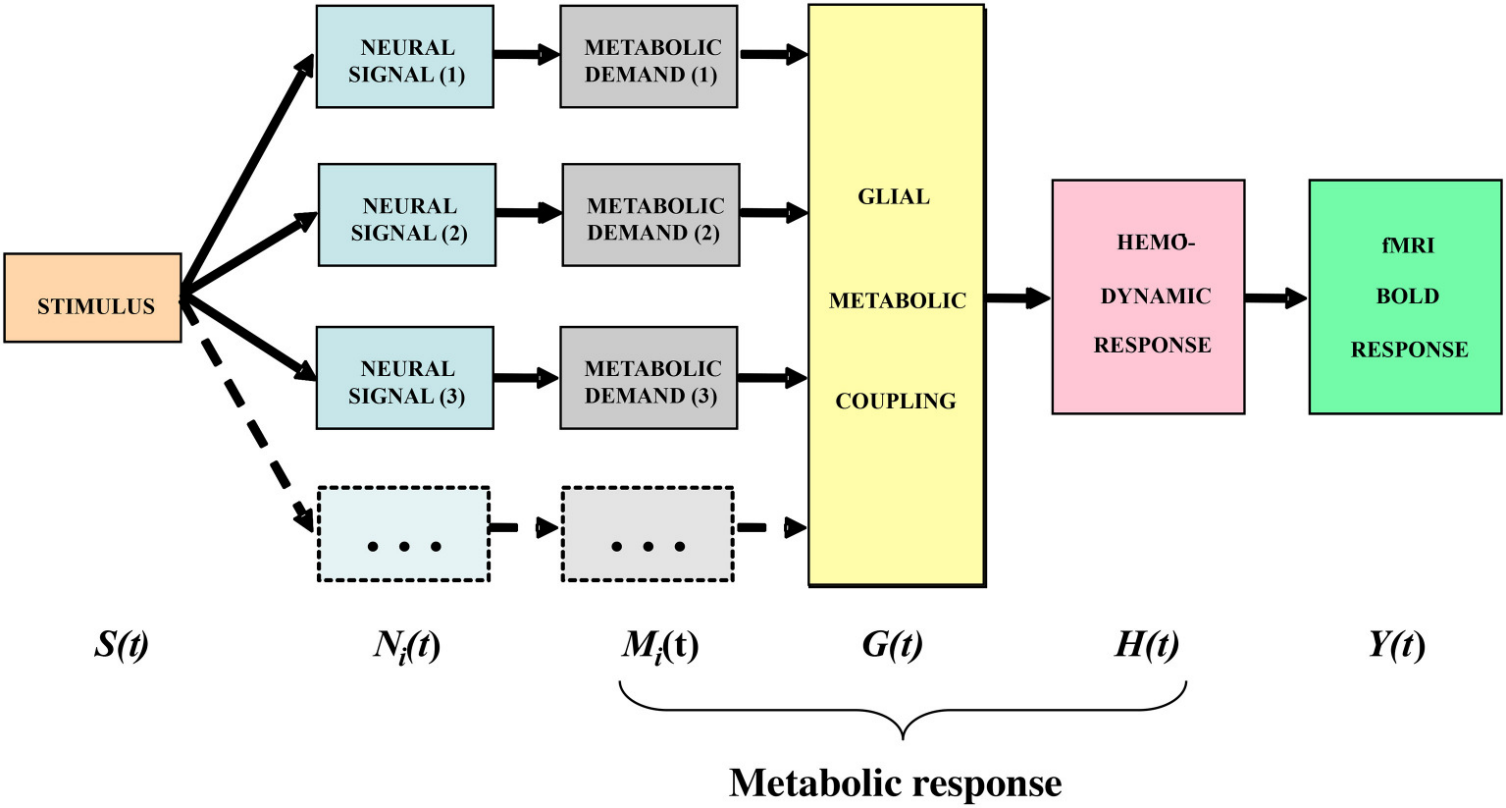
**Block diagram of the main processing stages that lead up to the BOLD signal**. The subscript *i* indicates that the stage incorporates multiple components from homogeneous subpopulations within a voxel (from Tyler and Likova, 2011, 2014).

In general terms, the stimulus impinging on the subject generates a sequence of neural responses starting with the transduction into a neural signal within the sensory receptors, which then propagates to the brain and activates various populations of neurons within the voxels then being analyzed by the fMRI technique. For instance, the signals arriving from the retina generate synaptic activation of the populations of cortical cells, which generates a local energetic demand for the restoration of the neurotransmitter molecules carrying the activation signals. The chain of cortical metabolic processing, illustrated in the block diagram of Figure 3, progresses from the local metabolic demand generated by the neural events at the synapse through the metabolic coupling mediated by the neighboring astrocyte glial cells as a whole to the processes of oxygen delivery by the adjacent capillaries that is detected by the imaging methodology. It is important to emphasize that the astrocyte metabolic processes are slow relative to the intracellular signal dynamics, about as slow as the processes of hemodynamic oxygen supply. The time constant of the astrocyte responses is known to be of the order of several seconds (Kelly and Van Essen, 1974; Filosa et al., 2004; Metea and Newman, 2006; Schummers et al., 2008), and it is clear that there must be a substantial pre-hemodynamic component from these slow responses. Kelly and Van Essen (1974) and Schummers et al. (2008) also show that the slower glial responses are as strongly tuned to local stimulus orientation as are the neural responses, implying a tight functional coupling between them. However, at present too little is known of their dynamics and/or nonlinearities to securely assign precise time constants to the astrocytic component relative to the hemodynamic component.

### Specifying the model framework

The model framework is slightly modified from that in Tyler and Likova (2011). We treat the neural responses within each voxel for a given stimulus *S(t)* as generated by sets of homogeneous populations with similar signal waveforms *N*_*i*_*(t)* within each population (see Figure 3). For generality, it is assumed that these neural signal waveforms are generated by a nonlinear transduction from the input stimulus. The transduction from each neural population response to the local metabolic demand *M*_*i*_*(t)* is further assumed for generality to be nonlinear. The overall metabolic demands *G(t)* within a voxel are met primarily by the surrounding astrocytes, which support the required neural energy consumption over time and space and make a complementary metabolic demand *G(t)* on the adjacent vasculature. This integrated metabolic demand stimulates the vascular hemodynamic processes *H(t)* provide the requisite oxygen and glucose exchange to replenish the energy depletion in the astrocytes. The last three stages constitute the metabolic response that determines the ratio of oxygenated to deoxygenated hemoglobin in the blood complement of a given voxel that is estimated through the paramagnetic reaction as the BOLD signal *Y(t)*. These post-neural processing stages are often modeled as a linear metabolic response kernel (*mrk*) convolved with the presumed neural signal.

The terms of the conceptual model in Figure 3 are related by a series of mathematical operations specified in Table 1 (modified from Tyler and Likova, 2011). The three operators are: (i) linear convolution (⊗), a nonlinear amplitude relation (*f[ ]*), and a multiple linear integrator (Σ). Note that each stage of the model is treated as the linear convolution of the output signal from the previous stage with a temporal response kernel designated by lower case initial for the respective process, i.e., the neural response function *n(t)*, the metabolic response function *m(t)*, the glial response function *g(t)*, the true hemodynamic response function *h(t)*, the paramagnetic response function *p(t)* that generates the BOLD signal, and an approximate metabolic response kernel *mrk(t)*. This last process corresponds to a linear approximation of the metabolic coupling relation implied by the previous three stages. The linear integration across multiple parallel elements within the voxel provided by the glial coupling stage corresponds to a nonlinear process in the context of single-channel solution.

**Table 1:** **Mathematical model of the operations involved in the generation of the BOLD signal from the input stimulus**.

**Output**	**Generation logic**	**Features**
Neural signal	*N_*i*_(t) = f[S(t)* ⊗ *n_*i*_(t)]*.*e*^−*t*∕*γ*^	Nonlinear transducer with adaptation
Neural metabolic demand	*M_*i*_(t) = f[N_*i*_(t)* ⊗ *m(t)]*	Nonlinear transducer
Glial metabolic coupling	*G(t) = ΣM_*i*_(t)* ⊗ *g(t)*	Multiple linear summation
Hemodynamic response	*H(t) = G(t)* ⊗ *h(t)*	Linear (slow)
Paramagnetic response	*BOLD(t) = H(t)* ⊗ *p(t)*	Linear (fast)
Metabolic coupling relation	*BOLD(t)* ≈ *ΣM_*i*_(t)* ⊗ *mrk(t)*	Combines 4 previous stages

#### Nonlinearities

Unlike the example in Figure 2, however, typical LFP responses show a much weaker transient at offset than onset (see **Figure 6**, column 1), which implies the presence of an adaptation process decreasing the transient component over time. Such adaptation can be readily modeled by the nonlinear process of an exponential decay with time constant γ multiplying the response over time (after convolution with the stimulus), as shown in the first line of Table 1.

Table 1 thus invokes three kinds of nonlinearities in the overall model—an amplitude nonlinearity (lines 1 and 2), an adaptive temporal nonlinearity (the exponential term in line 1), and a multiple summatory nonlinearity (line 3). Nevertheless, these stages are typically inaccessible, therefore for practical purposes, they are approximated by the linear model form in the last line of the table: a function representing the neural metabolic demand evoked by the neural response to the stimulus presentation is convolved with the metabolic response kernel (*mrk*).

### Nonlinear model of the local field potential (LFP)

#### The neural signal

A comprehensive model of the BOLD therefore requires an accurate model of the intracellular potential dynamics coupled to stimulation. If the excitatory and inhibitory transmitter release are symbolized by ψ_*e*_, ψ_*i*_, we can specify the relationships between the synaptic input and the intracellular potential *V*_*I*_*(t)* as follows:

(1) $$\begin{array}{l} V_{I}\left(t\right)=\sum_{\eta_{e}}\psi_{e}(t)-\sum_{\eta_{i}}\psi_{i}(t)+n_{0}\left(0,\sigma_{0}\right)\\ \;=stim(t)⊗\left(\eta_{e}t^{k_{e}}e^{-t/\tau_{e}}-\eta_{i}t^{k_{i}}e^{-t/\tau_{i}}\right)+n_{0}\left(0,\sigma_{0}\right) \end{array}$$

where η_*e*_ and η_*i*_ are the number of excitatory and inhibitory transmitter molecules, respectively (or, strictly, the number of ionic charges carried by the net inflow of transmitter molecules per unit time) and *n*(0, σ_*I*_) is the cumulated noise of the intracellular signal from quantal, thermal, and transmitter sources.

To avoid complications, we do not specify the contributory components of the intracellular noise. For example, the quantal component will decrease in standard deviation as luminance level is increased, and the transmitter source may decrease in standard deviation as the activation level decreases, but we assume the totality of noise sources add up to a constant Gaussian noise source to a first approximation. This assumption has been evaluated in detail by Carandini (2004) in coupled intracellular and extracellular recordings. His model provides an accurate quantitative account of the strong signal-dependence of the variability of the extracellular spike rate (Tolhurst et al., 1981; Vogels et al., 1989) in terms of a purely additive Gaussian intracellular noise passing through the threshold-like nonlinearity of the spike generation process. Thus, the additive Gaussian noise assumption for the intracellular signal governing the metabolic demand is fully compatible with the signal-dependent properties of neural spike noise.

The constants η_*e*_ and η_*i*_ are specified for every individual cell and will vary substantially among cell types. Indeed, they will vary substantially with the placement of the intracellular (e.g., patch-clamp) recording site in relation to the synaptic inputs of the cell. However, for the present purposes, the relevant values are the average values integrated over large volumes of cortex leading to the local metabolic demand that underlies the BOLD signal, as reflected in the local field potential (LFP) recorded at a site in the extracellular medium.

As is highlighted by the data of Figure 2, there are adaptive effects in the neural response with a complex time course that can be approximated by two exponentials with time constants of about 1 s and >30 s, respectively. These are remarkably prolonged neural processes on the time scale of the recorded BOLD signal from the same general region of cortex (blue trace) as indicated by the fact that the recorded LFP signal does not fully match the predicted BOLD activation (red curve). The negative LFP signal in Figure 2 following stimulus offset has a similar (but inverted) time course to that following the stimulus onset, implying that the adaptation effect is a subtractive inhibition rather than solely a multiplicative form of fatigue (which would have no negative rebound). If such a gain control were purely multiplicative, the amplitude of signal change at offset would be substantially less than that at onset, whereas the two amplitudes are similar within about 10%. Thus, the adaptive inhibition must be predominantly subtractive rather than multiplicative gain control and may correspond to the tonic intracellular hyperpolarization suggested by Carandini and Ferster (1997, 2000) to be the mechanism for pattern adaptation. However, it is adapting essentially to a dynamic input modulation, and hence the sustained LFP signal should be treated as deriving from a full-wave rectified transform of the intracellular potential.

Formally, the neural signal for the present analysis is considered to be the extracellular voltage *V*_*j*_*(t)* in each *j*th subpopulation of neurons with homogeneous response characteristics and is related to the intracellular voltage according to

(2) $$\begin{array}{l} V+\tau_{j}\frac{dV}{dt}=\alpha_{j}V_{I},\text{ where }V=V_{j}\left(t-\Delta t\right) \end{array}$$

and where τ_*j*_ and ζ_*j*_ are the time constants of the two exponentials, Δ*t* is an onset delay, and α_*j*_ is a scaling factor, for a given neural population *j*.

Solving Equation (2) for *V*_*j*_(*t*) and restricting it to positive *t* gives:

(3) $$\begin{array}{l} V_{j}(t-\Delta t)=\frac{\alpha_{j}}{\tau_{j}}V_{I}(t-\Delta t)⊗e^{-(t-\Delta t)/\tau_{j}},t>\Delta t\\ \;=0,\;t<\Delta t \end{array}$$

Thus, the neural input for the contributions of the various neural populations to the LFP for the model of Table 1 is:

(4) $$\begin{array}{l} n\left(t\right)=\underset{j>1}{\sum }V_{j}\left(t\right) \end{array}$$

together with a sustained component given by:

(5) $$n_{1}(t)\;=\int v_{1}(t)$$

Finally, the *mrk* for the metabolic coupling relation in the last line of Table 1 is assumed to be a gamma function of the form:

(6) $$\begin{array}{l} mrk\left(t\right)=\alpha_{M}t^{k}\cdot e^{-t∕\tau_{M}} \end{array}$$

where α_*M*_, *k*, and τ_*M*_ are the characteristic constants of the *mrk* dynamics.

**Figure 4:**
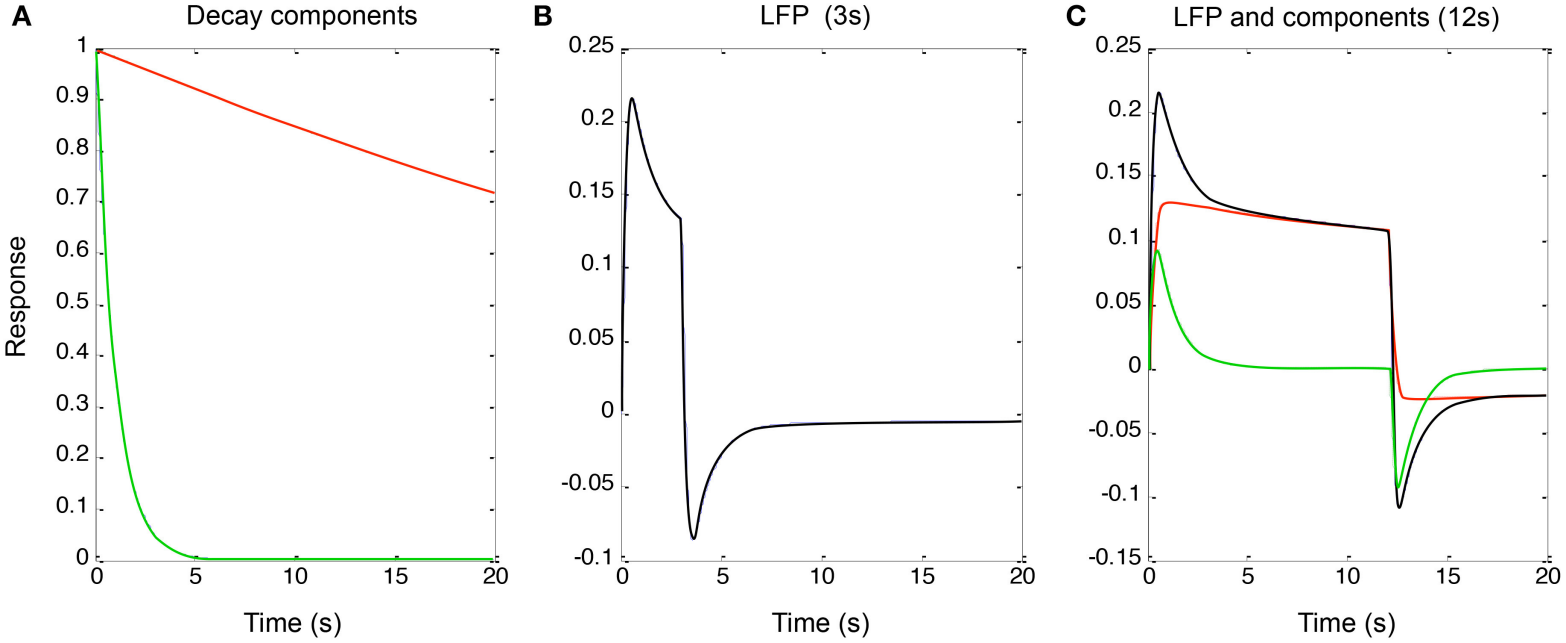
**(A)** Two exponential decay processes (red and green curves) used to account for the adaptation effects in Figure 2. **(B)** The properties of Equation (4) for a stimulus of 3 s duration. **(C)** The same for 12 s duration (black curve), together with the components making it up (red and green curves). (See text for details).

To implement the additive (parallel-process) model of Equation (3) (shown in Figure 4 for a qualitative fit to the data of Figure 2), the two decay components had time constants of 1 and 60 *s* (“slow” and “fast” components, red and green curves in Figure 4A). These processes were convolved with a neural signal derived from sum of the two components after convolution of the two components with the rectangular form of the continuous stimulus for 3 and 12 s, the latter corresponding to the responses in Figure 2. This model captures the qualitative features of the LFP data (Figures 4B,C, black curves) with the sum of the two component responses (red and green curves in Figure 4C). Again, it is difficult to obtain such a combination of the two component slopes with purely serial model, because this would imply a convolution of the two exponentials which would necessarily result in a function dominated by the slower process rather than allowing both processes full expression.

### Neurometabolic coupling

**Figure 5:**
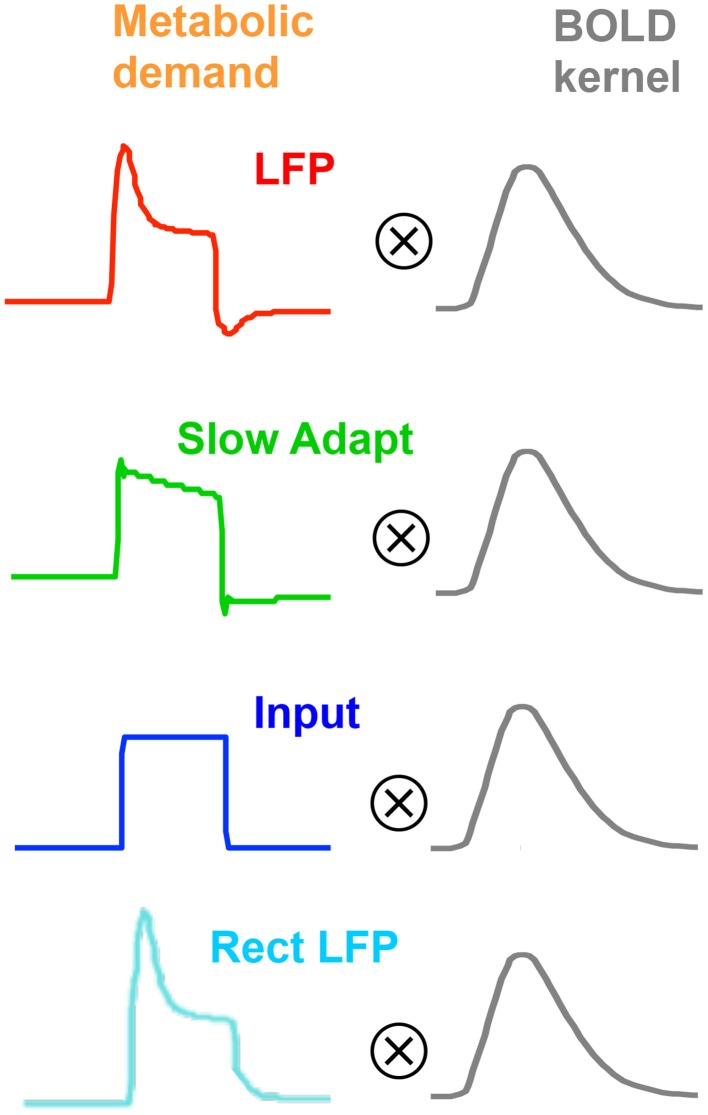
**Four plausible models of the BOLD response characteristic through linear convolution with various metabolic response kernel hypotheses**.

As will become evident, we will need a range of models of neurometabolic coupling to account for the variety of data available. We therefore develop four options as to what aspect of the neural signal is coupled through the metabolic demand to the BOLD response (see Figure 5). All four options assume that the coupling to generate the BOLD response can be approximated as a linear process of convolution with the *mrk* (last line of Table 1), with the nonlinearities occurring in terms of the predominant aspect of the neural signal and the early stages of the metabolic chain that is assumed to be driving the BOLD response. Thus, the coupling of the *mrk* with a LFP model response is assumed to be linear (as in Friston et al., 1998).

#### LFP coupling

The first model option (Figure 5, top row) is the original concept that the LFP represents the net neural signal in the voxel, which generates the metabolic demand that drives the metabolic recovery processes through in the blood supply (Lippert et al., 2010), as mediated by the intervening glial cells. The net neural signal contributing to the LFP is the input for a given cortical area as well as its local intracortical processing, including the activity of excitatory and inhibitory interneurons and the effect of neuromodulatory pathways (Logothetis, 2003, 2008; Magri et al., 2012). The LFP model for this option is specified in the first line of Table 1, which incorporates a slow adaptive process in addition to the fast and slow decay components of Equation (4).

#### Slow adaptive coupling

Instead of assuming that the MRK input derives from the whole LFP, it may be assumed to be specific primarily to the *slow adaptive component* of the model (Figure 5, second row), with the fast component attributable to spiking activity, which would have little impact on the BOLD response due it its low metabolic requirements (Logothetis, 2002, 2003, 2008; Logothetis and Wandell, 2004). Thus, the *mrk* is assumed to be solely the sustained component of Equation (5) followed by the adaptive process of line 1 of Table 1.

#### Neurotransmitter input coupling

An alternative option is the assumption that all the observed LFP adaptation is a function of extracellular signal diffusion *after* the metabolic demand has been defined by the neurotransmitter processes (Figure 5, third row). Under this assumption, the neurometabolic coupling would be with a *non-adaptive* sustained neurotransmitter response to the input signal, as proposed by Logothetis (2002, 2003, 2008) and specified in Equation (5). In particular, this hypothesis implies that there would be no transient off-response component contributing to the BOLD signal.

#### Rectified LFP coupling

A final option (Figure 5, fourth row) is that any deviation of the LFP from zero (either positive or negative) is mediated by the release of some form of neurotransmitter and represents a metabolic demand (Sotero and Trujillo-Barreto, 2007; Tyler and Likova, 2011), as specified in Equation (1) with η_*i*_ taking the value of −1. This assumption implies that the release of any neurotransmitter in the form of either excitatory or inhibitory synaptic coupling would constitute a neurometabolic load that generated a positive neurometabolic demand. A simplified model of such a demand would thus be represented by a rectified version of the nonlinear LFP (Rect LFP), although it is possible that this would still underestimate the metabolic demand due to electrical cancellation of the positive and negative components in different parts of the cell. Nevertheless, the rectified LFP would constitute a lower bound of the neurometabolic demand, and in particular would convey its characteristic of having no negative aspects. This simplified model can therefore be used as an initial assay of whether the rectification approach has merit, with possible elaboration if it provides a better fit than the other models.

## Methods

**Figure 6:**
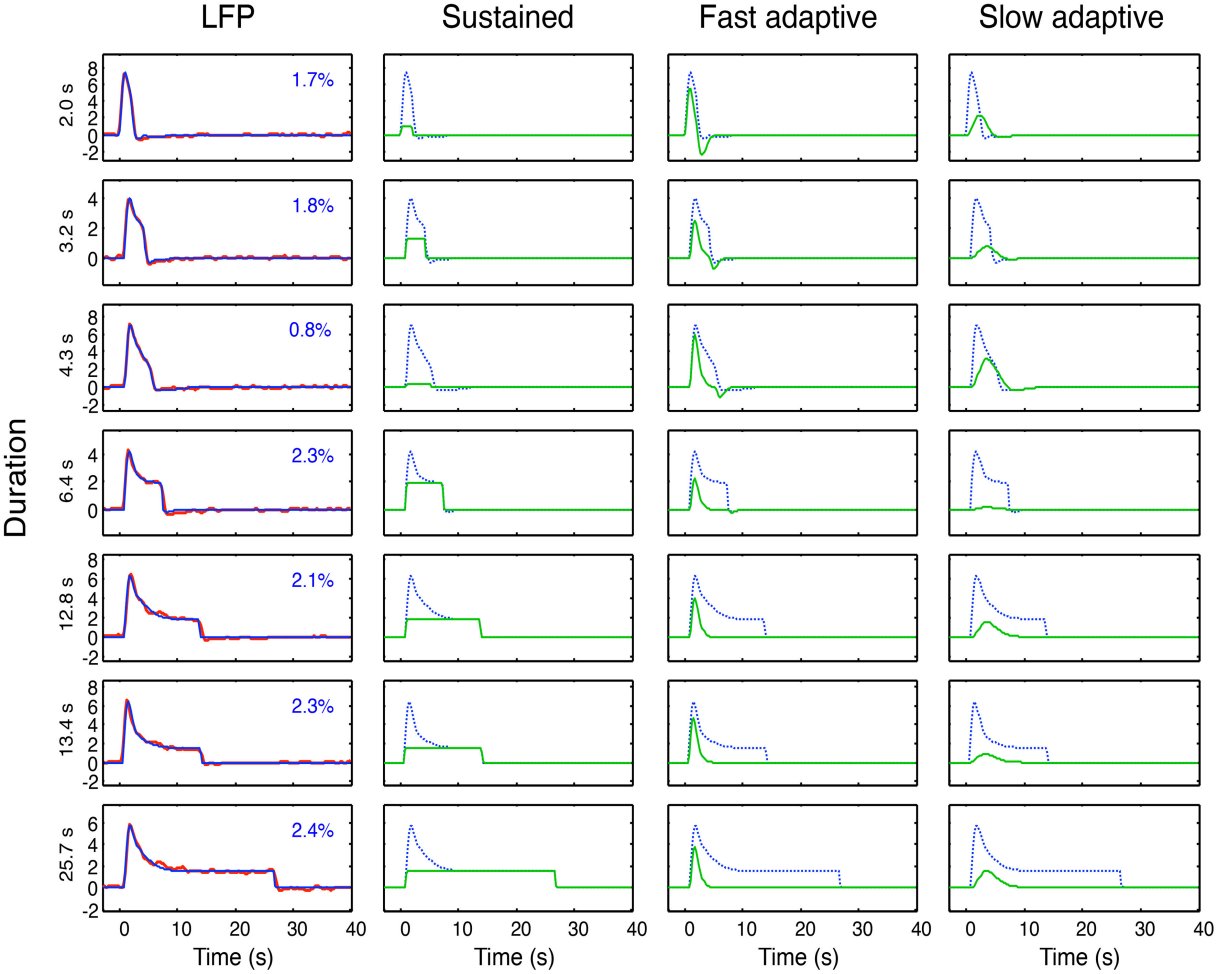
**Left column: Overall neural model fits (blue curves) to the average LFP responses (red curves) at each of 7 durations**. Proportion of variance unaccounted for (R^2^) shown as insets. Three right columns: Optimized sustained, fast and slow adaptive components (green curves for each duration) required to provide the overall neural model fits (blue curves). Note that residual variance (1–*R*^2^) is less than 3% in all cases, and must be considered to have fully characterized the LFP dynamics of V1.

As specified in the previous section, these four hypothetical forms of coupling have all been proposed in the literature. Here we may now compare their performance within primary visual cortex (V1) of macaque monkeys from LFP data made available to us by Nikos Logothetis from the study described in Figure 2 (see Logothetis, 2003, for details), with seven recording durations (2, 3.2, 4.3, 6.4, 12.8, 13.4, and 25.7 s). The LFP bandwidth was 10–300 Hz. The stimuli were large-field rotating checkerboards, alternating in direction every 2 s, designed to avoid response adaptation as much as possible. There were a total of 28 datasets, which are averaged for each available duration to provide the average data for the seven durations shown in Figure 6.

The model fitting was implemented through the Matlab fminsearch function for optimization of a parametrized function to data, with the mean squared error as the variable to be minimized. For the full LFP model of Equation (4), we needed to include a sustained (non-adaptive) component (Equation 5) in addition to the two adaptive components (see line 1 of Table 1) in order to capture the characteristics of the response; thus *i* = 1, 2. To fit the LFP model of Equation (4) to these data, the four dynamic parameters of τ_*i*_, ζ_*I*_, their onset delay Δ*t* and their adaptation time constant γ, were optimized for the fit to the mean responses simultaneously across all seven durations, together with amplitude of each component as a free parameter at each duration, making a total of 4 + 7 = 11 free parameters. For each duration, *n* = 64 and the residual variances for the LFP fits are specified in each panel of the first column of Figure 6. Thus the 64 × 7 = 448 parameters of the average LFP data are fit with a model of 11 free parameters. The component weights of the resulting three components (green curves) are shown in the remaining columns of Figure 6, with the overall LFP waveforms (dashed blue curves) for comparison.

For the full model fits to the BOLD waveforms, the optimized LFP fit for each duration was convolved with an *mrk* according to Equation (6), with *k* and τ_*M*_ optimized to all durations simultaneously, together with an amplitude parameter α_*M*_ and baseline shift parameter for each of the 7 durations (2+2^*^7 = 16 free parameters). Since the BOLD sampling rate was 250 ms, the dataset of 160 × 7 = 1120 parameters was being fit with the 16 free parameters for each of the four models of metabolic demand shown in Figure 5 (given the LFP fit as the input function for each duration). The presence of 160 samples at each duration implies that individual fits are significant at *p* < 0.001 of the *F*-test, providing Bonferroni correction to *p* < 0.02 for multiple applications to 16 fits if they account for more than 61% of the variance (i.e., if the residual variance is less than 39% of the overall variance).

Moreover, for the ratio between any two variances to be significant, the ratio has to exceed 1.63 on the *F*-test for significance at *p* < 0.001 (which provides an appropriate level of Bonferroni correction for the test validity at *p* < 0.05 over the multiple applications of 6 pairwise comparisons among the 4 models, times 7 durations, or a total of 42 test applications).

## Modeling results

The first aspect of the study was to fit the model of Equation (4) to the average LFPs across duration, as shown in Figure 6. This model fit had the twofold goal of (a) providing a low-free-parameter characterization of the LFP waveform and of (b) defining its component structure in terms of the components developed in Equations (2–5) and Figure 5. The specific model components were thus a sustained component matching the stimulus input, a fast adaptive component and a slower adaptive component. (Note that the adaptation gives the latter two components a much reduced offset transient relative to their onset transients at long durations; Figure 6, columns 3 and 4.) The optimal dynamic parameters are specified in Table 2.

**Table 2:** **Optimal parameter values for the LFP model**.

**Parameter**	**Δ*t***	**τ_*i*_**	**ζ**	**γ**
Value (ms)	0.61	1.01	2.79	2.95

The neural model fits to the LFP waveforms show that the three-component model has the appropriate structure to match all the evident features of the waveform, accounting for an average of 98% of the variance. Except at short durations, all three components are approximately equally weighted in the combined model. It might be possible to capture the data with the same component weights across duration, but the goal of the study is not LFP modeling *per se*, so it was not relevant to pursue this issue.

**Figure 7:**
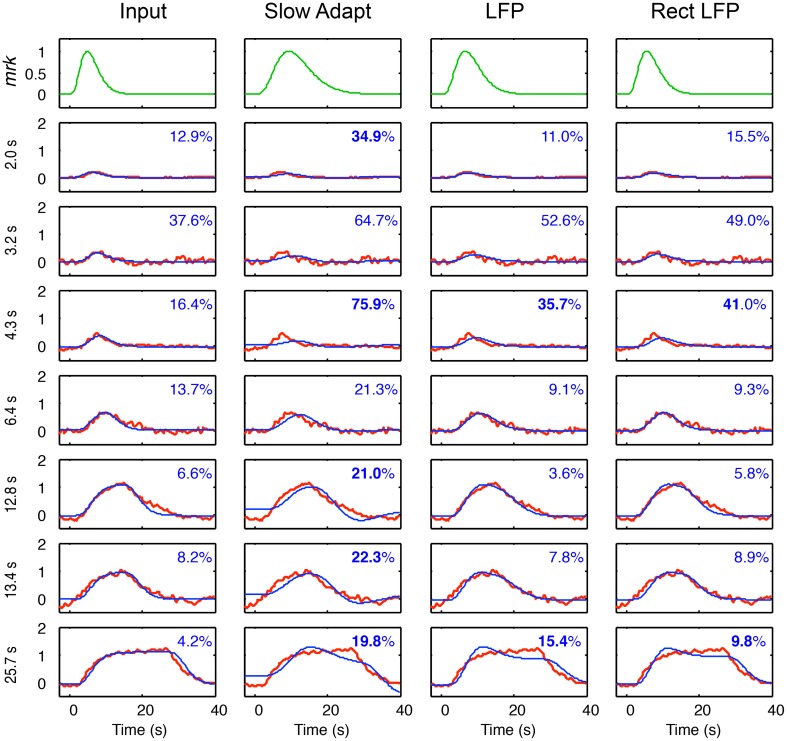
**Model fits (blue curves) for the four models of BOLD coupling for the respective components of the LFP coupling models of Figure 6 fitted to the average BOLD responses (red curves) at 7 durations**. Top row shows the BOLD metabolic kernels (green curves) required to best fit for each model. Insets specify the percent residual variance of the fits, with those significantly higher than those for the Input model shown in bold. The non-adapting neural Input model thus provides the best fit to the average BOLD data overall.

Fits of the four models for the metabolic demand to the BOLD responses at each duration are shown in Figure 7, based on the components of the LFP fits in Figure 6, together with their optimized *mrk* (top row). Note that the BOLD *mrk* parameters in Figure 7 were allowed to vary across the four models (as there is no prior on the relationships among the models), but held constant over the 7 stimulus durations, as the metabolic parameters are not expected to be affected by the nature of the stimulus. The time constants of the optimized *mrk* waveforms in terms of peak latency were 4.8, 9.3, 6.6, and 2.7 s for the four models, respectively, based on a 5th-order gamma function model.

As specified in Methods, the individual fits are significant at *p* < 0.03 if the residual variance is < 39%. Thus, all the fits are significant except for several of those for the 3.2 and 4.3 s durations.

For the specific comparisons among the different models, the statistically significant cases may be assessed as any having ratio of the residual variances greater than 1.63 between model fits at a given stimulus duration, as described in Methods.

Across the durations, each of the model fits is significantly worse than for the Input model at a few durations (residual variances shown in bold), particularly those for the Slow Adapt model, and no model has significantly better fits than the Input model at any duration, with the exception of the LFP model at one duration—12.8 s (Figure 7). At the longest duration, the Input model fits are significantly better those for all three other models. Thus, taken together, the net result is that the Input model provides the best fit overall across the 7 stimulus durations.

## Discussion

Taking all durations into account, the results of this modeling study imply that the BOLD response is most closely coupled with the neurotransmitter input waveform defined by the sustained response close to the boxcar waveform of the stimulus time course, without the transients and adaptive nonlinearities exhibited by the LFP waveform. The best-fitting BOLD *mrk* was a 5th-order gamma function with a peak time of 4.8 s and no inhibitory rebound, accounting for more than 90% of the variance at the three longest durations (which would correspond to correlations between the model and the data of >0.95). In practice, of course, the inputs to V1 voxels would have passed through several stages of neural processing in the visual pathway, including transmission delays, and temporal integration, but these effects are evidently too small to be resolved on the time scale of the available analysis. Also, it should be noted that the initial transients characteristic of most neuronal responses are specifically minimized by the design of the stimuli, which provided continuous movement alternating in direction every 2 s, and hence that the initial neural response should be expected to closely match the stimulus specification. In this context, it is actually surprising to find the LFP exhibiting the pronounced initial transient that is evident in Figure 6, since the stimulus was specifically designed to minimize such deviations from the input boxcar waveform in the form of directional adaptation. However, the present data and model fits imply that any longer-term adaptation to this kind of motion stimulus is happening beyond the stage of the neural inputs to V1, as there is no tendency on average for the BOLD response to decline at the longest durations, and hence it must derive from a non-adapting component of the neural response in V1.

Thus, the net conclusion from this study agrees with that of (2002, 2003, and 2008), that the form of the BOLD signal is most compatible with the input to the neuronal response, i.e., with the energetics of the primary neural activation that requires a glutamatergic metabolic response. It is noteworthy that this is the coupling that involves the briefest estimated *mrk*, because this is the metabolic demand with the least transient input of the four. In fact, the *mrk* peak for this case is occurring at only 4.8 s, a fairly typical value for the general understanding for human BOLD responses. (Note, however, that this value cannot be compared directly with the HRF of the standard approach, as the HRF incorporates all preceding neural dynamics, whereas the *mrk* is restricted to the metabolic response kernel by the assumptions of the analysis.)

Moreover, the model *mrk* had no delay parameters. As can be seen from the examples in Figure 7 (first column), there is no visible tendency for the rise of the BOLD onset to lag the model fits. This result suggests that there is no inherent BOLD delay relative to the gamma-function model of the *mrk* in relation to neural activation beyond that implied by the order of the gamma function required to account for the full BOLD waveform. Any further delays that may be needed in a range of GLM analyses of the gamut of tasks in the literature may be attributed to neural processing delays.

It should be emphasized that the linear convolution of the *mrk* stage required for the present fits implies (although it does not prove) that any further complexity or cortical diversity of the measured BOLD dynamics, as reported by Fox et al. (2005), Handwerker et al. (2012) or Likova and Tyler (2007), for example, is attributable to variations in the underlying neural signals rather than to variations in the BOLD HRF *per se*. On this basis, the results further imply that the use of stimuli that allow neural adaptation prior to arrival in the cortex, and hence an adaptive waveform for the cortical input (wherever in the cortex that may be), would show an adaptive BOLD response in that region of cortex. Moreover, a neural input that had a negative rebound in the signal arriving at the cortex would show a negative rebound in the BOLD response. For example, the rotating noise stimulus of the Logothetis study analysis here was changed in direction every 2 s to minimize adaptation effects. If instead it had been maintained indirection for the full 40 s time period, classic motion adaptation would have been expected during the stimulus presentation, with a negative rebound corresponding to the motion aftereffect. Such behavior was indeed reported by Tootell et al. (1995). Evidence in favor even stronger adaptation effects in a purely transient noise paradigm is provided by Likova and Tyler (2007).

## Conclusion

The good quality of the full model fits to the combined LFP and BOLD data as a function of duration provides a principled assessment of the nature of the neural/BOLD coupling behavior underlying BOLD fMRI and provides structured insights into the nature of the neural signal components contributing to the BOLD response dynamics. In general, the results are consistent with previous work employing a linear convolution of the stimulus waveform with a gamma-function model of the BOLD dynamics, but they provide further insight into the nature of the underlying processes involved. In particular, they reveal that no negative rebound of the BOLD response is required to account for the recorded BOLD waveforms.

In relation to the first stage of the model process, the extremely high quality of the model fits to the LFP data provides strong evidence that the LFP component model has the appropriate component structure to account for the mechanisms contributing to the recorded LFP dynamics. This question was not the focus of the present paper, but we note that there are surprisingly few modeling studies attempting to characterize the mechanisms of neural response dynamics, particularly in the case of LFPs, and propose this model structure as the starting point for more targeted studies of this issue.

In relation to the question of assessing the neural signals contributing to BOLD responses throughout the brain, a key tool in this enterprise is an accurate model structure for the likely neural responses in any local volume of cortex. The parameters of such a model can allow for optimization to the range of responses encountered across stimulus conditions, cortical regions and individual brains. The success of the present analysis helps to provide validation that this is an achievable goal, and should encourage similar efforts for a wider range of stimulus conditions to determine how far the present model can be generalized and what other aspects need to be included to characterize the full range of such constraints.

### Conflict of interest statement

The authors declare that the research was conducted in the absence of any commercial or financial relationships that could be construed as a potential conflict of interest.
